# Association between menstrual cycle phase and metabolites in healthy, regularly menstruating women in UK Biobank, and effect modification by inflammatory markers and risk factors for metabolic disease

**DOI:** 10.1186/s12916-023-03195-w

**Published:** 2023-12-08

**Authors:** Kirstin A. MacGregor, Frederick K. Ho, Carlos A. Celis-Morales, Jill P. Pell, Iain J. Gallagher, Colin N. Moran

**Affiliations:** 1 Physiology, Exercise and Nutrition Research Group, University of Stirling, Stirling, Scotland, UK; 2 School of Health and Wellbeing, University of Glasgow, Glasgow, Scotland, UK; 3 School Cardiovascular and Metabolic Health, University of Glasgow, BHF Glasgow Cardiovascular Research Centre, Glasgow, Scotland, UK; 4 Human Performance Lab, Education, Physical Activity and Health Research Unit, University Católica del Maule, Talca, Chile; 5 Centre for Biomedicine and Global Health, School of Applied Sciences, Edinburgh Napier University, Sighthill Campus, Sighthill Court, Edinburgh, UK

**Keywords:** Glucose, Lipid, Luteal phase, Follicular phase, Metabolic control, Triglyceride

## Abstract

**Background:**

Preliminary evidence demonstrates some parameters of metabolic control, including glycaemic control, lipid control and insulin resistance, vary across the menstrual cycle. However, the literature is inconsistent, and the underlying mechanisms remain uncertain. This study aimed to investigate the association between the menstrual cycle phase and metabolites and to explore potential mediators and moderators of these associations.

**Methods:**

We undertook a cross-sectional cohort study using UK Biobank. The outcome variables were glucose; triglyceride; triglyceride to glucose index (TyG index); total, HDL and LDL cholesterol; and total to HDL cholesterol ratio. Generalised additive models (GAM) were used to investigate non-linear associations between the menstrual cycle phase and outcome variables. Anthropometric, lifestyle, fitness and inflammatory markers were explored as potential mediators and moderators of the associations between the menstrual cycle phase and outcome variables.

**Results:**

Data from 8694 regularly menstruating women in UK Biobank were analysed. Non-linear associations were observed between the menstrual cycle phase and total (*p* < 0.001), HDL (*p* < 0.001), LDL (*p* = 0.012) and total to HDL cholesterol (*p* < 0.001), but not glucose (*p* = 0.072), triglyceride (*p* = 0.066) or TyG index (*p* = 0.100). Neither anthropometric, physical fitness, physical activity, nor inflammatory markers mediated the associations between the menstrual cycle phase and metabolites. Moderator analysis demonstrated a greater magnitude of variation for all metabolites across the menstrual cycle in the highest and lowest two quartiles of fat mass and physical activity, respectively.

**Conclusions:**

Cholesterol profiles exhibit a non-linear relationship with the menstrual cycle phase. Physical activity, anthropometric and fitness variables moderate the associations between the menstrual cycle phase and metabolite concentration. These findings indicate the potential importance of physical activity and fat mass as modifiable risk factors of the intra-individual variation in metabolic control across the menstrual cycle in pre-menopausal women.

**Supplementary Information:**

The online version contains supplementary material available at 10.1186/s12916-023-03195-w.

## Background

The prevalence of impaired metabolic control is increasing in pre-menopausal women [1]. Impaired metabolic control is typically characterised by decreased insulin sensitivity, fasting hyperglycaemia and dyslipidaemia [2]. Additionally, recent evidence indicates that glycaemic and lipidemic variability are integral components of metabolic control [3–6]. Dysregulation in parameters of metabolic control contributes to the pathophysiology of metabolic disorders, such as metabolic syndrome and type 2 diabetes (T2D) [7]. Therefore, it is crucial to examine factors that affect metabolic control in pre-menopausal women.

The menstrual cycle is a fundamental biological rhythm governing female physiology in pre-menopausal women. Regulated across an approximately 4-weekly duration, the menstrual cycle is characterised by cyclical fluctuations in pituitary hormones (luteinising hormone and follicle-stimulating hormone) and ovarian hormones (estradiol and progesterone) [8, 9]. Ovarian hormones exert regulatory roles within lipid and glucose homeostasis [10, 11]. Correspondingly, previous research has reported cyclical variation in parameters of metabolic control across the menstrual cycle, in association with fluctuations in ovarian hormone profiles [12–24]. However, findings are inconsistent; other studies report no effect of the menstrual cycle phase on parameters of metabolic control [25, 26]. These inconsistencies may be caused by the small sample sizes and heterogenous female populations recruited in these studies. Recent research demonstrates that variation in metabolic control across the menstrual cycle differs by categories of adiposity, cardiorespiratory fitness and physical activity [27]. However, further research is required to elucidate the role of these factors in the relationship between menstrual cycle phase and metabolites.

One mechanism which may contribute to the variation in metabolic control across the menstrual cycle is low-grade inflammation. Several inflammatory cytokines undergo rhythmic fluctuation across the menstrual cycle, including C-reactive protein (CRP), interleukin-4 (IL-4), insulin-like growth factor-1 (IGF-1) and tumour necrosis alpha (TNF-a) [25, 28–31]. Low-grade inflammation is positively associated with impaired metabolic control, including insulin resistance, hyperglycaemia and dyslipidaemia [25, 32–34]. Together, these are suggestive of inflammation being a potential mediator of the variation in metabolic control across the menstrual cycle; however, further research is needed to elucidate this role.

This study aimed to investigate the association between the menstrual cycle phase and metabolite concentrations. Following this, we aimed to explore whether adiposity, fitness, physical activity and inflammatory markers mediate and/or moderate these associations.

## Methods

### Study protocol

UK Biobank is a large prospective, population-based study which recruited 502,682 participants between March 2007 and December 2010 [35]. Individuals aged 37–73 years living within a 10-mile radius of 1 of 22 assessment centres across England, Scotland and Wales were invited to participate by post (5.5% response rate [36]). During the baseline assessment visit, participants undertook a self-completed touch-screen questionnaire, a brief computer-assisted interview, physical and functional measures and sampling of blood, urine and saliva, as described in detail elsewhere [35, 37]. The outcomes of interest in the current study were glucose; triglyceride; triglyceride and glucose index (TyG); total, HDL and LDL cholesterol; and total to HDL cholesterol. The exposure in this study was the menstrual cycle phase at the time of sampling.

### Participant inclusion criteria

**Fig. 1:**
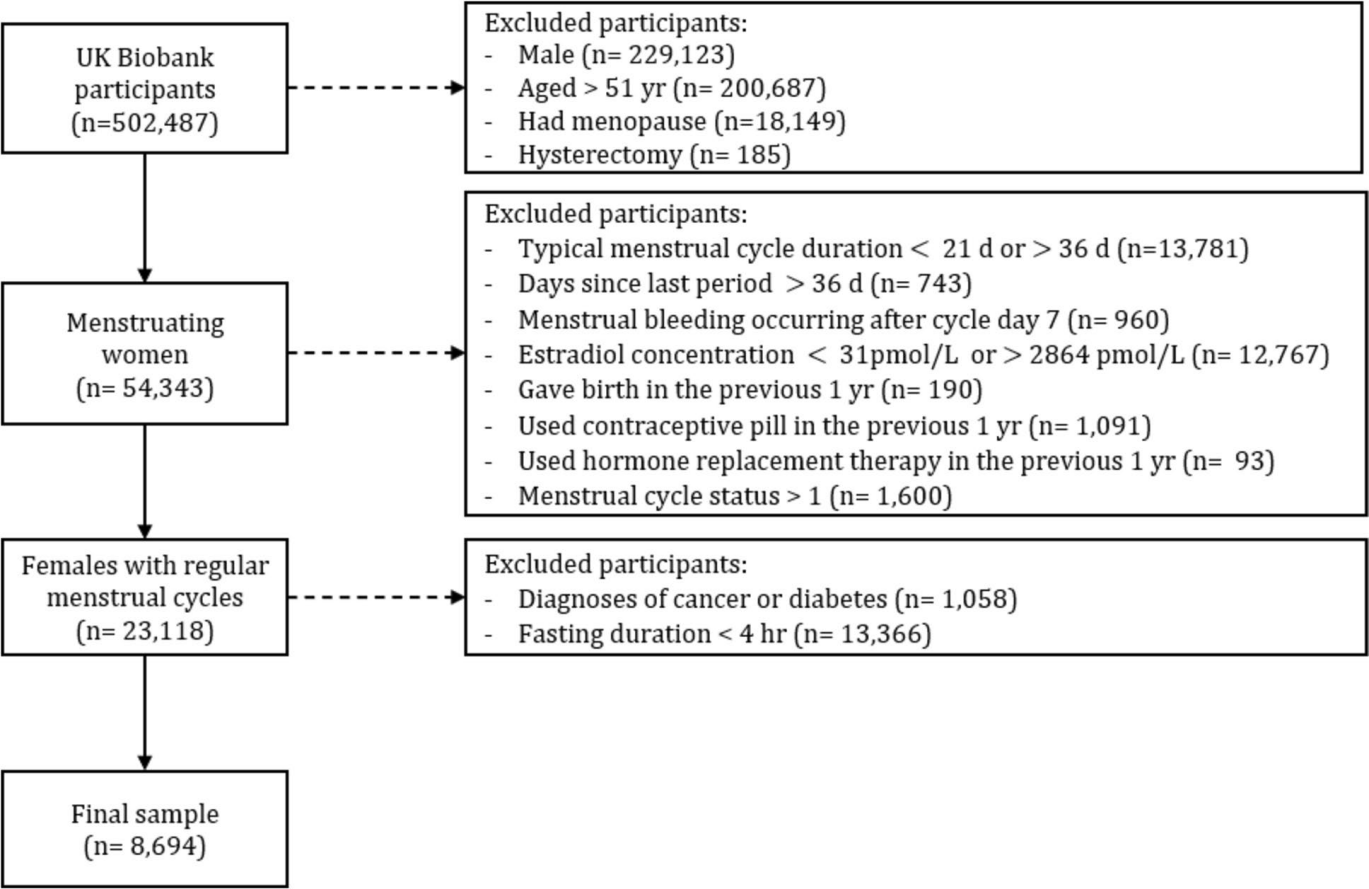
Flow diagram depicting participant inclusion in the current study

Menstrual cycle length was assessed in pre-menopausal women (Fig. 1); 66,447 participants answered the question “How many days since your last menstrual period?”. Women were excluded from the current analysis based on factors that alter hormonal concentrations across the menstrual cycle: menstrual cycle duration $<$ 21 or $>$ 36 days; > 36 days since last period; menstrual bleeding occurring after cycle day 7; estradiol concentration $<$ 31 pmol/L or $>$ 2864 pmol/L; $<$ 1 year since last gave birth; $<$ 1 year since last used contraceptive pill; or $<$ 1 year since last used hormone replacement therapy. Participants were further excluded if they reported T2D or cancer diagnoses at baseline assessment. Blood samples were not collected following an overnight fast; therefore, participants were excluded from analysis if they recorded a fasting time $\le$ 4 h.

### Exposure variables

The menstrual cycle phase was assessed using self-reported answers to the questions “How many days since your last menstrual period?” and “How many days is your usual menstrual cycle?”. Each participant provided responses to these questions at one timepoint. To account for the non-uniformity of participant menstrual cycle lengths, standardised time within the menstrual cycle was calculated relative to each participant using the formula: (days since last menstrual period/days in usual menstrual cycle) whereby 0 represents the start of the menstrual cycle and 1 represents the end of the menstrual cycle. This corresponds to the approximate phases: follicular phase 0.00–0.54 and luteal phase 0.54–1.00 [38]. At this point, participants were further excluded from the current study if their menstrual cycle phase was greater than 1.

### Outcome variables

The blood sampling protocol and biochemical analysis have previously been outlined and validated elsewhere [39, 40]. Serum concentrations of glucose; triglyceride; total, HDL and LDL cholesterol; and CRP were assessed using the AU5800 (Beckman Coulter, CA, USA). Serum concentration of estradiol was assessed using the DXI 800 (Beckman Coulter, CA, USA). Serum concentration of IGF-1 was assessed using the Liaison XL (DiaSorin, Saluggia, Italy). Serum concentration of glycated haemoglobin (HBA1c) was assessed using the Variant II Turbo (BioRad, CA, USA). Manufacturers’ analytical ranges for these analytes were as follows [41, 42]: glucose 0.6–45 mmol/L, triglyceride 0.1–11.3 mmol/L, total cholesterol 0.5–18.0 mmol/L, HDL cholesterol 0.05–4.65 mmol/L, LDL cholesterol 0.26–10.3 mmol/L, CRP 0.08–80.00 mg/L, estradiol 72–17,621 pmol/L, IGF-1 1.3–195.0 nmol/L and HBA1_c_ 15–184 mmol/mol. The TyG index was calculated as (Ln[(triglyceride mg/dL) × fasting glucose mg/dL)/2]) [43]. Total cholesterol to HDL ratio (total to HDL cholesterol mmol/L) was calculated as (total cholesterol mmol/L)/(HDL cholesterol mmol/L).

### Confounding variables

Age was calculated from self-reported date of birth and assessment date. Ethnicity was self-reported and categorised as white, black, Asian, Chinese, other or mixed. Area-level socioeconomic deprivation was assessed by the Townsend score based on self-reported home postcode [44].

### Potential mediators and moderators

#### Anthropometric measurements

Anthropometric measurements were obtained by trained personnel according to standard protocols and using calibrated equipment [37]. Height was measured without shoes using a Seca 202 height measure. Whole body mass, fat mass and fat-free mass were measured without shoes and outdoor clothing to the nearest 0.1 kg using the Tanita BC-418 MA body composition analyser. Body mass index (BMI) was calculated as mass in kilogrammes divided by the square of height in metres. Body composition was expressed as fat mass and fat-free mass in kilogrammes, both as absolute values and as percentage of total body mass. Quartiles of fat and fat-free mass percentage were derived.

#### Fitness

A subset of participants in UK Biobank underwent cardiorespiratory fitness testing, 1412 of whom were eligible for inclusion in this study. Cardiorespiratory fitness was assessed using a submaximal 6-min cycle ergometer test with workload adjusted for participant age, sex, height, weight and resting heart rate [45]. Heart rate was measured pre-, during and post-exercise via a four-lead electrocardiogram. Predicted maximal work rate was calculated by extrapolating heart rate and workload before exercise and at the end of exercise to predict maximal heart rate (208 − (0.7 × age year)) [46], assuming a linear relationship [47]. Maximal oxygen uptake was predicted using the standard equation for oxygen utilisation during cycle ergometry (metabolic equivalents (METs): 7 + (workload W × 10.8/body mass kg)/3.5 [47]. Grip strength was measured using a Jamar J00105 hydraulic hand dynamometer [48]. Isometric grip force was assessed from a 3-s maximal grip effort of the left- and right-side arms. The mean of the left- and right-side values was calculated and expressed in kilogrammes. Age-specific cardiorespiratory fitness and grip strength *z*-scores were derived.

#### Physical activity

Self-reported physical activity was assessed using the International Physical Activity Questionnaire (IPAQ). Data processing guidelines published by IPAQ were followed [49]. Time spent at each level of activity was weighted by metabolic energy equivalent (MET) (walking 3.3 METs, moderate intensity 4.0 METs, vigorous intensity 8.0 METs), then summated to calculate total MET hours per week of physical activity. Participants were assigned to low, moderate and high categories of physical activity based on standard data processing guidelines [49].

#### Menstrual cycle symptoms

Physical symptoms associated with the menstrual cycle, known as dysmenorrhea (abdominal discomfort, abdominal pain, menstrual cramps or other problems), were assessed via questionnaire responses. “Degree bothered by menstrual cramps or other problem with your period during the last 3 months” was self-reported as “not bothered at all”, “bothered a little” or “bothered a lot”. Discomfort or pain occurring only during menstrual cycle bleeding during the last 3 months was self-reported as “yes” or “no”.

#### Fasting duration

Time since the last meal or drink, excluding plain water, was self-reported and recorded in hours.

### Statistical analyses

All data analyses were conducted using R version 3.6.3 [50], together with the libraries: emmeans [51], lme4 [52], mgcv [53], tidymv [54] and tidyverse [55]. Sample sizes varied across the analyses due to missing data. Continuous variables were reported as mean and standard deviations and categorical variables were reported as the number of observations and their respective percentage.

To assess the relationship between the menstrual cycle phase and metabolites (glucose; triglyceride; TyG index; total, HDL and LDL cholesterol; and total to HDL cholesterol), spline model fitting was conducted using generalised additive models (GAM) [53]. A cyclic cubic regression spline was fitted to the menstrual cycle phase, which constrains the start and end points of the smooth to the same value.

#### Mediator analyses

To identify factors as potential mediators of the relationship between the menstrual cycle phase and metabolites, we first examined the association between metabolite concentration and potential mediators. Factors considered as potential mediators were BMI, fat mass %, fat-free mass %, total physical activity, grip strength, cardiorespiratory fitness, CRP and IGF-1. The associations between metabolites and potential mediators were examined using GAMs fitted with a thin palate regression spline. To avoid over-fitting the parts of the distribution with low N, the top 1% of values were removed for CRP and IGF-1 (CRP > 16.23 mg/L, IGF-1 >$37.9$ mmol/L). To investigate whether the associations between the menstrual cycle phase and metabolites were mediated by these factors, separate GAM models fitted with a cyclic cubic regression spline were run that included each potential mediator as a continuous covariate. To test whether the inclusion of the covariate improved model fit, an *F*-test was conducted.

#### Moderator analyses

To investigate whether the associations between menstrual cycle phase and metabolites differed by anthropometric, physical activity and fitness factors, potential moderators were coded as quartiles and included as an interaction term in the GAM models fitted with a cyclic cubic regression spline. Where multiple measures for a similar physiological parameter were available, only one measure from each physiological parameter was selected for moderator analysis to avoid multicollinearity. For anthropometric variables, the indices most sensitive to body composition were selected: fat mass (%) and fat-free mass (%). For physical activity levels, IPAQ total physical activity categories were selected because this index summates walking, moderate and vigorous physical activity levels and the categories reflect current physical activity recommendations [49]. The significance of model fit was tested for each category of the sub-groups investigated. To test whether the inclusion of the interaction term improved the model fit, an *F*-test was conducted.

In all GAM analyses, the smoothing parameter was estimated by the generalised cross-validation method. Non-linearity was tested using likelihood ratio tests. All GAM analyses were adjusted for confounding factors (age, ethnicity and deprivation). A *p*-value < 0.05 was considered statistically significant in all analyses.

#### Sensitivity analyses

To examine whether the semi-fasted nature of blood samples affected the results, sensitivity analysis was conducted by adjusting all GAM models for fasting duration. Additionally, one-way ANOVAs were conducted to examine whether metabolite concentrations differed by reported fasting duration, categorised in hourly increments from 0 to 8 h. Pairwise comparisons were conducted with Tukey adjustment after significant ANOVA results. Additional sensitivity analysis was conducted to examine if any behavioural or physiological effects of menstrual cycle symptoms affected results by restricting the cohort to women who did not report any menstrual cycle symptoms.

All scripts used for analysis and figure generation can be found at https://github.com/kirstin-macgregor/MCmetab_Biobank.

## Results

### Participant characteristics

The women in this cohort were predominantly (89.5%) of white ethnicity, with a mean age of 45 years (SD 3 years); 48.3% were normal weight (Table 1). As expected, some participant characteristics (weight, BMI, % and absolute fat mass, % fat-free mass, walking, vigorous and total physical activity, grip strength, cardiorespiratory fitness, estradiol, CRP and IGF-1) demonstrated non-linear relationships with menstrual cycle phase (Additional file 1: Table S1).

**Table 1:** Participant characteristics

Characteristic	Mean ± SD or *n* (%)
Age (years)	44.9 ± 2.8
Weight (kg)	70.4 ± 14.3
Height (m)	1.6 ± 0.1
BMI (kg/m^2^)	26.2 ± 5.2
Fat mass (kg)	25.0 ± 10.3
Fat mass (%)	34.3 ± 7.2
Fat-free mass (kg)	45.4 ± 5.0
Fat-free mass (%)	27.0 ± 3.7
Nutritional status	
Under-weight, BMI $<$ 18.5	75 (0.9)
Normal-weight, BMI $\le$ 18.5 to $<$ 25	4203 (48.3)
Over-weight, BMI $\le$ 25 to $<$ 30	2800 (32.2)
Obese, BMI $\ge$ 30	1590 (18.3)
Ethnicity	
White	7782 (89.5)
Black	123 (1.4)
Mixed	228 (2.6)
Asian	321 (3.6)
Chinese	67 (0.7)
Other	150 (1.7)
Physical activity	
Walking (MET min/week)	1050.9 ± 1093.2
Moderate (MET min/week)	774.0 ± 1049.1
Vigorous (MET min/week)	664.4 ± 1022.8
Summed (MET min/week)	2489.4 ± 2405.3
Grip strength (kg)	26.9 ± 6.0
Cardiorespiratory fitness (METs)	9.6 ± 2.4
Estradiol (pmol/L)	560.1 ± 383.8
IGF-1 (mmol/L)	23.8 ± 5.4
CRP (mg/L)	2.0 ± 3.6
HBA1c (mmol/mol)	33.0 ± 3.6
Glucose (mmol/L)	4.78 ± 0.49
Triglyceride (mmol/L)	1.20 ± 0.66
TyG index (mmol/L)	4.50 ± 0.23
Total cholesterol (mmol/L)	5.37 ± 0.90
HDL cholesterol (mmol/L)	1.55 ± 0.34
LDL cholesterol (mmol/L)	3.27 ± 0.71
Total to HDL cholesterol (mmol/L)	3.59 ± 0.93

*BMI* body mass index, *CRP* C-reactive protein, *HBA1c* glycated haemoglobin, *HDL* high-density lipoprotein, *IGF-1* insulin-like growth factor-1, *LDL* low-density lipoprotein, *TyG index* triglyceride to glucose index. Continuous variables are reported as mean ± 1 SD for continuous variables and categorical variables are reported as the number of observations and their respective percentage

### Associations between menstrual cycle phase and metabolites

Menstrual cycle phase was associated with total cholesterol (estimated degrees of freedom (EDF) 3.4, *p* < 0.001), HDL cholesterol (EDF 2.9, *p* < 0.001), LDL cholesterol (EDF 3.6, *p* = 0.012) and total to HDL cholesterol (EDF 5.4, *p* < 0.001) (Table 2). No significant associations were observed between the menstrual cycle phase and glucose (EDF 1.3, *p* = 0.072), triglyceride (EDF 4.0, *p* = 0.066) or TyG index (EDF 3.6, *p* = 0.100). Phase-specific metabolite values for the early follicular phase, ovulatory phase and mid-luteal phase are presented in Additional file 1: Table S2.

**Table 2:** Associations between menstrual cycle phase and metabolite concentration menstrual cycle phase

Variable (mmol/L)	*N*	EDF	Dev exp	*p*-value	Minimum (mmol/L (MC phase))	Maximum (mmol/L (MC phase))	Variation (mmol/L (%))
Glucose	7780	1.3	0.68	0.072	4.77 (0.46)	4.79 (0.96)	0.02 (0.43)
Triglyceride	8653	4.0	1.81	0.066	1.18 (0.92)	1.24 (0.67)	0.06 (5.32)
TyG index	7778	3.6	2.84	0.100	4.50 (0.96)	4.52 (0.67)	0.02 (0.45)
Total cholesterol	8651	3.4	2.30	** < 0.001**	5.32 (0.88)	5.44 (0.25)	0.12 (2.27)
HDL cholesterol	7779	2.9	1.88	** < 0.001**	1.53 (0.88)	1.59 (0.46)	0.06 (3.85)
LDL cholesterol	8638	3.6	1.59	**0.012**	3.25 (0.88)	3.32 (0.21)	0.07 (2.15)
Total to HDL cholesterol	7776	5.4	1.46	** < 0.001**	3.50 (0.50)	3.65 (0.13)	0.15 (4.17)

*P*-values represent the significance level for smoothed terms in the GAM. Boldface text denotes a significant *p*-value (< 0.05). Analyses were adjusted for age, ethnicity and deprivation. Menstrual cycle (MC) phase values are shown on a scale of 0–1; this corresponds to the approximate phases: follicular phase, 0–0.54; luteal phase, 0.54–1 [38]

*Dev exp* deviance explained in percentage, *EDF* estimated degrees of freedom, *HDL* high-density lipoprotein, *LDL* low-density lipoprotein, *TyG index*, triglyceride to glucose index

### Association between anthropometric, lifestyle, fitness and inflammatory markers and metabolites

BMI, fat mass %, fat-free mass %, cardiorespiratory fitness, CRP and IGF-1 were associated with all metabolites (all *p* < 0.01) (Table 3). Grip strength was associated with glucose, triglcyeride, HDL, total to HDL cholesterol and TyG index (all *p* < 0.01), but non-significant associations were found for LDL (*p* = 0.166) and cholesterol (*p* = 0.096). Total physical activity was associated with total, LDL and HDL cholesterol; triglyceride; total to HDL cholesterol; and TyG index (all *p* < 0.05), but the association with glucose was non-significant (*p* = 0.413).

**Table 3:** Association between metabolites and anthropometric, lifestyle, fitness and inflammatory markers

Potential mediator	Metabolite (mmol/L)	*N* val	EDF	Dev exp	*p*-value
BMI (kg/m^2^)	Glucose	7548	1.86	1.20	** < 0.001**
	Triglyceride	8387	4.94	16.39	** < 0.001**
	TyG index	7546	4.45	19.37	** < 0.001**
	Total cholesterol	8385	3.34	3.88	** < 0.001**
	LDL	8372	4.26	7.28	** < 0.001**
	HDL	7547	4.40	16.08	** < 0.001**
	Total to HDL cholesterol	7544	5.07	21.22	** < 0.001**
Fat mass (%)	Glucose	7458	6.01	1.30	** < 0.001**
	Triglyceride	8289	4.29	14.60	** < 0.001**
	TyG index	7456	3.94	17.64	** < 0.001**
	Total cholesterol	8287	3.74	4.38	** < 0.001**
	LDL	8274	4.05	7.98	** < 0.001**
	HDL	7457	3.92	14.18	** < 0.001**
	Total to HDL cholesterol	7454	4.74	19.65	** < 0.001**
Fat-free muscle mass (%)	Glucose	7450	2.42	0.95	** < 0.001**
	Triglyceride	8281	5.67	13.48	** < 0.001**
	TyG index	7448	4.09	16.38	** < 0.001**
	Total cholesterol	8279	3.95	4.59	** < 0.001**
	LDL	8266	4.47	8.06	** < 0.001**
	HDL	7449	3.98	12.56	** < 0.001**
	Total to HDL cholesterol	7446	4.41	18.05	** < 0.001**
Grip strength (kg)	Glucose	7550	1.00	0.55	** < 0.001**
	Triglyceride	8388	2.41	1.99	** < 0.001**
	TyG index	7548	1.71	2.85	** < 0.001**
	Total cholesterol	8386	1.00	1.94	0.166
	LDL	8373	1.35	1.39	0.096
	HDL	7549	2.24	1.65	**0.002**
	Total to HDL cholesterol	7546	2.48	1.45	** < 0.001**
Cardiorespiratory fitness (METs)	Glucose	1505	1.00	3.24	** < 0.001**
	Triglyceride	1601	4.61	9.72	** < 0.001**
	TyG index	1505	2.77	12.65	** < 0.001**
	Total cholesterol	1602	2.32	4.17	** < 0.001**
	LDL	1602	4.09	5.90	** < 0.001**
	HDL	1506	2.59	7.99	** < 0.001**
	Total to HDL cholesterol	1505	4.17	11.68	** < 0.001**
Summed PA (MET min/week)	Glucose	6413	5.11	0.58	0.413
	Triglyceride	7130	3.89	2.91	** < 0.001**
	Total cholesterol	7128	2.38	2.11	**0.004**
	TyG index	6411	4.99	3.79	** < 0.001**
	LDL	7117	3.26	2.05	** < 0.001**
	HDL	6411	4.28	2.81	** < 0.001**
	Total to HDL cholesterol	6408	4.36	2.90	** < 0.001**
CRP (mg/dL)	Glucose	7572	1.00	0.66	**0.001**
	Total cholesterol	8409	5.49	3.10	** < 0.001**
	Triglyceride	8411	7.40	12.57	** < 0.001**
	LDL	8396	6.12	4.14	** < 0.001**
	HDL	7571	4.57	8.72	** < 0.001**
	Total to HDL cholesterol	7568	6.42	11.97	** < 0.001**
	TyG index	7570	7.30	14.68	** < 0.001**
IGF-1 (mmol/L)	Glucose	7572	2.69	0.91	** < 0.001**
	Triglyceride	8411	1.00	2.24	** < 0.001**
	TyG index	7570	1.00	3.33	** < 0.001**
	Total cholesterol	8409	2.51	2.17	**0.003**
	LDL	8396	1.32	1.72	** < 0.001**
	HDL	7571	2.64	2.11	** < 0.001**
	Total to HDL cholesterol	7568	1.00	1.88	** < 0.001**

*P*-values represent the significance level for smoothed terms in the GAM. Boldface text denotes a significant *p*-value (< 0.05). Analyses were adjusted for age, ethnicity and deprivation

*CRP* C-reactive protein, *EDF* estimated degrees of freedom, *HDL* high-density lipoprotein, *IGF-1* insulin-like growth factor-1, *LDL* low-density lipoprotein, *TyG index* triglyceride to glucose index

### Anthropometric, lifestyle, fitness and inflammatory markers as potential mediators of the associations between metabolites and menstrual cycle phase

Anthropometric, physical activity, strength and fitness markers which were significantly associated with metabolite concentrations were next considered as potential mediators (Table 4). The associations between menstrual cycle phase and glucose, triglyceride and TyG index remained non-significant when including each of the mediator variables in the model (all *p* > 0.05), except the mediators BMI (*p* = 0.010), fat mass % (*p* = 0.026), fat-free mass % (*p* = 0.036), cardiorespiratory fitness (*p* = 0.015), CRP (*p* = 0.008) and IGF-1 (*p* = 0.004) for the outcome variable triglyceride, and the mediators BMI (0.022), cardiorespiratory fitness (0.012), CRP (*p* = 0.009) and IGF-1 (*p* = 0.003) for the outcome variable TyG index. Menstrual cycle phase was associated with total, LDL and total to HDL cholesterol following the inclusion of the mediator variables BMI, fat mass %, muscle mass %, summed PA and IGF-1 in the model (all *p* < 0.01), but not cardiorespiratory fitness (*p* = 0.292). Menstrual cycle phase was associated with HDL following the inclusion of each of the mediator variables (*p* < 0.05). Model fit was improved by the inclusion of each of the mediator variables for all metabolites (*p* < 0.001), except for the mediator grip strength for the outcome variable glucose and the mediator physical activity for the outcome variable LDL cholesterol. The inclusion of each of the mediator variables increased the percentage of deviance explained in the models for all metabolites. Phase-specific metabolite values for the early follicular phase, ovulatory phase and mid-luteal phase are presented in Additional file 1: Table S3.

**Table 4:** Effect of anthropometric, lifestyle, fitness and inflammatory markers on the association between metabolite concentration with menstrual cycle phase

Variable (mmol/L)	Potential mediator	*N* val	EDF	Dev exp	Minimum (mmol/L (MC phase))	Maximum (mmol/L (MC phase))	Variation (mmol/L (%))	Model *p*-value	Interaction *p*-value
Glucose	BMI (kg/m^2^)	7754	1.26	1.47	4.77 (0.46)	4.79 (0.96)	0.02 (0.40)	0.080	** < 0.001**
	Fat mass (%)	7657	1.32	1.67	4.77 (0.50)	4.79 (0.00)	0.02 (0.43)	0.070	** < 0.001**
	Fat-free muscle mass (%)	7649	1.35	1.32	4.77 (0.50)	4.79 (0.96)	0.02 (0.44)	0.065	** < 0.001**
	Grip strength (kg)	7757	1.31	0.72	4.77 (0.46)	4.79 (0.96)	0.02 (0.43)	0.074	0.320
	Cardiorespiratory fitness (METs)	1540	3.61	4.12	4.83 (0.42)	4.93 (0.71)	0.10 (2.04)	**0.030**	** < 0.001**
	CRP (mg/dL)	7759	1.27	1.06	4.77 (0.46)	4.79 (0.96)	0.02 (0.40)	0.086	** < 0.001**
	IGF-1 (mmol/L)	7780	0.00	1.01	4.78 (0.50)	4.78 (0.96)	0.00 (0.00)	0.532	** < 0.001**
Triglyceride	BMI (kg/m^2^)	8627	4.30	16.23	1.17 (0.88)	1.25 (0.63)	0.08 (6.35)	**0.010**	** < 0.001**
	Fat mass (%)	8522	3.99	14.63	1.17 (0.88)	1.23 (0.63)	0.06 (5.08)	**0.026**	** < 0.001**
	Fat-free mass (%)	8514	4.02	13.55	1.17 (0.88)	1.23 (0.63)	0.06 (4.97)	**0.036**	** < 0.001**
	Summed PA (MET min/week)	7328	0.34	2.92	1.20 (0.96)	1.20 (0.50)	0.01 (0.42)	0.296	** < 0.001**
	Grip strength (kg)	8628	3.96	2.10	1.18 (0.92)	1.24 (0.67)	0.06 (5.13)	0.079	** < 0.001**
	Cardiorespiratory fitness (METs)	1638	3.53	10.89	1.14 (0.88)	1.26 (0.58)	0.12 (9.99)	**0.015**	** < 0.001**
	CRP (mg/dL)	8629	3.73	12.14	1.17 (0.92)	1.23 (0.63)	0.06 (5.31)	**0.008**	** < 0.001**
	IGF-1 (mmol/L)	8653	4.07	2.39	1.16 (0.92)	1.24 (0.63)	0.08 (6.69)	**0.004**	** < 0.001**
TyG index	BMI (kg/m^2^)	7752	4.00	19.67	4.49 (0.92)	4.52 (0.63)	0.02 (0.54)	**0.022**	** < 0.001**
	Fat mass (%)	7655	3.72	18.06	4.50 (0.88)	4.51 (0.63)	0.02 (0.43)	0.062	** < 0.001**
	Fat-free mass (%)	7647	3.72	16.82	4.50 (0.88)	4.52 (0.63)	0.02 (0.42)	0.083	** < 0.001**
	Summed PA (MET min/week)	6581	0.85	4.04	4.50 (0.00)	4.51 (0.54)	0.01 (0.12)	0.209	** < 0.001**
	Grip strength (kg)	7755	3.50	3.06	4.50 (0.96)	4.52 (0.67)	0.02 (0.43)	0.108	** < 0.001**
	Cardiorespiratory fitness (METs)	1540	3.51	13.91	4.49 (0.96)	4.54 (0.63)	0.05 (1.07)	**0.012**	** < 0.001**
	CRP (mg/dL)	7757	3.71	14.42	4.49 (0.96)	4.52 (0.63)	0.02 (0.54)	**0.009**	** < 0.001**
	IGF-1 (mmol/L)	7778	3.77	3.64	4.49 (0.96)	4.52 (0.63)	0.03 (0.64)	**0.003**	** < 0.001**
Total cholesterol	BMI (kg/m^2^)	8625	3.43	4.27	5.31 (0.88)	5.45 (0.25)	0.14 (2.52)	** < 0.001**	** < 0.001**
	Fat mass (%)	8520	3.30	4.78	5.31 (0.88)	5.45 (0.25)	0.14 (2.59)	** < 0.001**	** < 0.001**
	Fat-free mass (%)	8512	3.26	5.02	5.31 (0.88)	5.45 (0.25)	0.14 (2.58)	** < 0.001**	** < 0.001**
	Summed PA (MET min/week)	7326	3.42	2.51	5.31 (0.88)	5.44 (0.25)	0.14 (2.59)	** < 0.001**	**0.001**
	Cardiorespiratory fitness (METs)	1639	0.43	4.40	5.39 (0.88)	5.41 (0.38)	0.02 (0.36)	0.292	** < 0.001**
	CRP (mg/dL)	8627	3.39	3.40	5.32 (0.88)	5.45 (0.25)	0.13 (2.41)	** < 0.001**	** < 0.001**
	IGF-1 (mmol/L)	8651	3.30	2.50	5.31 (0.88)	5.45 (0.25)	0.13 (2.45)	** < 0.001**	**0.001**
LDL cholesterol	BMI (kg/m^2^)	8612	3.59	7.59	3.25 (0.83)	3.33 (0.21)	0.09 (2.63)	**0.001**	** < 0.001**
	Fat mass (%)	8507	3.50	8.29	3.24 (0.83)	3.34 (0.21)	0.09 (2.81)	** < 0.001**	** < 0.001**
	Fat-free mass (%)	8499	3.44	8.40	3.24 (0.83)	3.34 (0.21)	0.09 (2.79)	** < 0.001**	** < 0.001**
	Summed PA (MET min/week)	7315	3.49	2.33	3.24 (0.88)	3.32 (0.21)	0.08 (2.40)	**0.007**	NA
	Cardiorespiratory fitness (METs)	1639	0.00	5.90	3.27 (0.92)	3.27 (0.42)	0.00 (0.00)	0.953	** < 0.001**
	CRP (mg/dL)	8614	3.66	4.20	3.25 (0.88)	3.33 (0.21)	0.08 (2.48)	**0.003**	** < 0.001**
	IGF-1 (mmol/L)	8638	3.62	1.94	3.24 (0.88)	3.33 (0.21)	0.08 (2.57)	**0.003**	** < 0.001**
HDL cholesterol	BMI (kg/m^2^)	7753	2.77	16.48	1.53 (0.92)	1.59 (0.46)	0.05 (3.35)	** < 0.001**	** < 0.001**
	Fat mass (%)	7656	2.66	14.57	1.53 (0.96)	1.59 (0.46)	0.05 (3.31)	** < 0.001**	** < 0.001**
	Fat-free mass (%)	7648	2.63	12.97	1.53 (0.96)	1.59 (0.46)	0.05 (3.35)	** < 0.001**	** < 0.001**
	Summed PA (MET min/week)	6581	2.59	3.18	1.54 (0.88)	1.59 (0.42)	0.06 (3.72)	** < 0.001**	** < 0.001**
	Grip strength (kg)	7756	2.94	2.11	1.53 (0.88)	1.59 (0.46)	0.06 (3.88)	** < 0.001**	**0.001**
	Cardiorespiratory fitness (METs)	1541	2.95	8.89	1.57 (0.79)	1.63 (0.38)	0.06 (3.91)	**0.026**	** < 0.001**
	CRP (mg/dL)	7758	3.07	9.02	1.53 (0.88)	1.59 (0.46)	0.05 (3.37)	** < 0.001**	** < 0.001**
	IGF-1 (mmol/L)	7779	3.55	2.41	1.54 (0.83)	1.59 (0.46)	0.05 (3.29)	** < 0.001**	** < 0.001**
Total to HDL cholesterol	BMI (kg/m^2^)	7750	5.35	21.75	3.52 (0.50)	3.67 (0.13)	0.15 (4.19)	** < 0.001**	** < 0.001**
	Fat mass (%)	7653	5.83	20.20	3.52 (0.50)	3.69 (0.13)	0.17 (4.80)	** < 0.001**	** < 0.001**
	Fat-free mass (%)	7645	6.08	18.61	3.51 (0.50)	3.69 (0.13)	0.18 (4.98)	** < 0.001**	** < 0.001**
	Summed PA (MET min/week)	6578	4.34	3.25	3.50 (0.50)	3.62 (0.13)	0.12 (3.47)	**0.002**	** < 0.001**
	Grip strength (kg)	7753	5.69	1.77	3.50 (0.50)	3.65 (0.13)	0.15 (4.21)	** < 0.001**	** < 0.001**
	Cardiorespiratory fitness (METs)	1540	3.36	12.60	3.45 (0.38)	3.58 (0.67)	0.13 (3.58)	0.170	** < 0.001**
	CRP (mg/dL)	7755	5.72	12.03	3.51 (0.50)	3.66 (0.13)	0.14 (3.99)	**0.001**	** < 0.001**
	IGF-1 (mmol/L)	7776	6.29	2.22	3.51 (0.50)	3.66 (0.67)	0.15 (4.12)	**0.002**	** < 0.001**

Model *p*-values represent significance for the smoothed term of the menstrual cycle phase in the GAM. Interaction p-value represents the F-test comparing model fit with and without inclusion of mediator. Boldface text denotes a significant *p*-value (< 0.05). Analyses were adjusted for age, ethnicity, deprivation and fasting duration. Menstrual cycle phase values are shown on a scale of 0–1; this corresponds to the approximate phases: follicular phase, 0–0.54; luteal phase, 0.54–1 [38]

*BMI* body mass index, *CRP* C reactive protein, *dev exp* deviance explained in percentage, *EDF* estimated degrees of freedom, *HDL* high-density lipoprotein, *IGF-1* insulin-like growth factor-1, *LDL* low-density lipoprotein, *TyG index* triglyceride to glucose index

### Anthropometric, lifestyle and fitness markers as potential effect modifiers of the association between menstrual cycle phase and metabolites

**Fig. 2:**
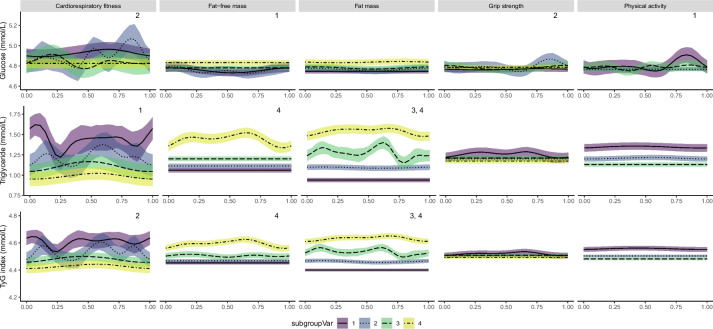
Variations in glucose, triglyceride and TyG index across the menstrual cycle for each model. Fat mass %, muscle mass %, age-specific grip strength (kg) *z*-score and age-specific cardiorespiratory fitness (METs) level *z*-score are categorised as quartiles. Physical activity (METs) is categorised into low, medium and high according to previously defined criteria. Curves represent GAM estimates using a smoothing spline function. Shaded areas represent 95% confidence intervals. The menstrual cycle phase is shown on a scale of 0–1 corresponding to the approximate phases: follicular phase, 0–0.54; luteal phase, 0.54–1 [38]. Analyses were adjusted for age, ethnicity and deprivation. Significant model fit for each sub-group is denoted by the respective number at the top right corner. TyG index, triglyceride to glucose index

**Fig. 3:**
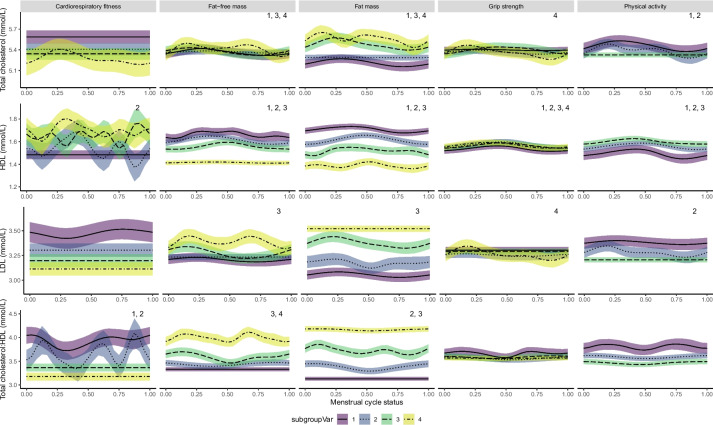
Variations in total cholesterol, HDL, LDL and total choleserol:HDL across the menstrual cycle for each model. Fat mass %, muscle mass %, age-specific grip strength (kg) z-score and age-specific cardiorespiratory fitness (METs) z-score are categorised as quartiles. Physical activity (METs) is categorised into low, medium and high according to previously defined criteria. Lines represent GAM estimates using a smoothing spline function. Shaded areas represent 95% confidence intervals. The menstrual cycle phase is shown on a scale of 0–1; this corresponds to the approximate phases: follicular phase, 0–0.54; luteal phase, 0.54–1 [38]. Analyses were adjusted for age, ethnicity and deprivation. Significant non-linear relationships for each category level are denoted by the respective number at the top right corner. LDL, low-density lipoprotein; HDL, high-density lipoprotein

The menstrual cycle phase was significantly associated with triglyceride; TyG index; total, LDL and HDL cholesterol; and total to HDL cholesterol in above median categories of fat mass and fat-free mass % (Figs. 2 and 3; Additional file 1: Table S4). In below median categories of fat mass, menstrual cycle phase was only significantly associated with total, HDL and total to HDL cholesterol. In below median categories of fat-free mass, menstrual cycle phase was significantly associated with glucose, total cholesterol and HDL. In low and/or medium categories of physical activity, menstrual cycle phase was significantly associated with glucose and total, HDL and LDL cholesterol. In the high physical activity category menstrual cycle phase was only significantly associated with HDL. In below median categories of cardiorespiratory fitness, the menstrual cycle phase was significantly associated with glucose, triglyceride, TyG index, HDL and total to HDL cholesterol. No consistent findings were observed when including categories of grip strength in models. The inclusion of the interaction terms improved the overall model fit for each of the sub-groups for all metabolites (*p* < 0.05). Phase-specific metabolite values for the early follicular phase, ovulatory phase and mid-luteal phase are presented in Additional file 1: Table S5.

### Sensitivity analyses

The severity of menstrual cycle symptoms is positively associated with variations in physical activity levels and inflammatory markers across the menstrual cycle [56, 57]. When we excluded women with any menstrual cycle symptoms (abdominal discomfort, abdominal pain, menstrual cramps or other problems with their menstrual cycle), the relationships between metabolites and menstrual cycle phase were generally similar in the mediator and sub-group analyses, except for lipids in some models (Additional file 1: Tables 6, 7 and 8). When we adjusted each GAM model for fasting duration, similar results were obtained for all metabolites in the mediator and sub-group analyses (Additional file 1: Table S9, S10 and S11).

## Discussion

In this population-based study using data on 8694 UK Biobank participants, we observed significant non-linear associations between menstrual cycle phase and total, HDL, LDL and total to HDL cholesterol, but not glucose, triglyceride or TyG index. The associations between the menstrual cycle phase and metabolite concentrations were not mediated by anthropometric, physical fitness, physical activity or inflammatory markers. In contrast, the inclusion of anthropometric, fitness, physical activity and inflammatory markers improved the model fit and increased the proportion of deviance explained by the models for metabolite concentrations. Sub-group analyses determined that the associations between menstrual cycle phase and metabolite concentrations were predominantly restricted to women with above median fat mass and fat-free muscle mass, low or medium levels of physical activity and below median cardiorespiratory fitness.

During the luteal phase of the menstrual cycle, circulating glucose is reduced [13, 18] and is accompanied by an increase in whole-body insulin resistance [18–24]. However, results are inconsistent [25, 26, 58, 59]. Our data do not provide evidence supporting variations in glucose or TyG index, markers of whole-body insulin sensitivity across the menstrual cycle, prior to including anthropometric, physical fitness, physical activity or inflammatory markers in the model. The women in this study had a higher BMI and age (26.2 ± 5.2 kg/m^2^; 44.9 ± 2.8 years) compared with some previous studies [13, 18–22, 24], which may have contributed to discrepancies in findings.

Significant non-linear associations with menstrual cycle phase were observed for cholesterol profiles, but not triglyceride concentration. Total, LDL and total to HDL cholesterol were highest in the early follicular phase and declined during the luteal phase, whereas HDL cholesterol reached a peak during the late-follicular phase. This finding was consistent with other studies that reported favourable lipid profiles in the mid-luteal phase and confirmed this pattern of rhythmicity existed in a larger population [12, 13, 16, 26]. Elevated circulating endogenous estradiol during the mid-luteal phase promotes the synthesis of very-LDL cholesterol and inhibits lipoprotein lipase, thereby reducing LDL cholesterol and increasing HDL cholesterol formation [60]. Thus, our findings are congruent with the reported effects of endogenous estradiol on lipoprotein metabolism.

Low-grade inflammation is involved in the development of insulin resistance [61–64] and accordingly is an independent risk factor associated with hyperglycaemia [63, 65], hyperinsulinemia [63, 66] and dyslipidaemia [62]. In agreement with previous studies, we observed significant associations between CRP and IGF-1 with glucose; TyG index; total, HDL and LDL cholesterol; and total to HDL cholesterol. When CRP or IGF-1 were included in the models, the observed associations between menstrual cycle phase and total, LDL, HDL and total to HDL cholesterol did not change. Therefore, we did not report evidence that CRP or IGF-1 mediates the association between the menstrual cycle phase and metabolite concentration.

High adiposity, low cardiorespiratory fitness and low physical activity levels are risk factors for impaired metabolic control [67, 68]. Accordingly, we observed BMI, fat mass, fat-free mass, grip strength, cardiorespiratory fitness and physical activity were significantly associated with all metabolites. However, we found no evidence that the associations between metabolites were mediated by anthropometric, fitness or physical activity levels.

Greater variation in metabolites across the menstrual cycle was generally observed in women with above-median fat mass and below-median physical activity and cardiorespiratory fitness. This was consistent with a recent study that reported greater variation in glucose and insulin resistance across the menstrual cycle in individuals with a high BMI (> 25 kg/m^2^), low physical activity levels (< 500 MET min/week) and low cardiorespiratory fitness (< 50th age-specific $\dot{\mathrm{V}}$ O_2max_ percentile) [69]. Consistent with these findings, others report that adiposity, physical activity and cardiorespiratory fitness are associated with increased intra-individual variability in glycaemic and lipidemic control in women [67, 68]. Notably, our findings extend this evidence base by demonstrating that these risk factors for metabolic disease moderate variation in metabolite concentration across the menstrual cycle. However, the precise mechanisms that underlie the observed differences in the magnitude of variation in metabolites across the menstrual cycle between categories of adiposity, physical activity and fitness remain to be determined.

The severity of menstrual cycle symptoms is positively associated with behavioural changes and inflammatory markers, including CRP [25, 56, 57]. Therefore, we conducted a sensitivity analysis excluding women with menstrual cycle symptoms from the cohort. In this analysis, similar observations were detected between menstrual cycle phase and metabolite concentration in all models.

Intra-individual glucose and lipid variations are independent risk factors for metabolic disease in healthy women [70] and those with T2D [4, 5]. We found significant non-linear associations between the menstrual cycle phase and lipid profiles, with a variation of 2.2–5.3% across the menstrual cycle. Additionally, the sub-group analysis determined that variations in glucose TyG index and lipid profiles across the menstrual cycle were increased in individuals with high-fat mass and low physical activity (up to 25% increase). These magnitudes of variation in metabolites across the menstrual cycle are greater than reported within-cycle phase variation (1–9%) [26] and analytical technical coefficients of variation (1–2%) [41]. Given the role of intra-individual variation in the onset and progression of metabolic disease, these findings identify physical activity and fat mass as potentially modifiable risk factors for the prevention and risk reduction of metabolic disease in pre-menopausal women.

Our study has several limitations. The cohort of UK Biobank participants included in this study were middle-aged (40–50 years). We ensured any perimenopausal women were omitted from the cohort by including irregular menstrual characteristics in the exclusion criteria. Additionally, women with estradiol values $<$ 31 pmol/L or $>$ 2864 pmol/L were excluded, as the perimenopausal period of the reproductive cycle is characterised by abnormally high or low ovarian estradiol production [71]. Nonetheless, findings from this study must be extrapolated to younger populations with caution. Fasting blood samples were not collected in UK Biobank. Therefore, any participant with a fasting duration of less than 4 h was excluded from the analysis. This cut-off threshold was selected based on evidence demonstrating semi-fasted (≥ 4 h) measures of blood glucose and lipids are not significantly different from and are closely correlated to fasted measures (≥ 8 h) [72–75]. In this study, differences in metabolite concentrations between consecutive hours of fasting were not detected above 4 h (Additional file 1: Table S12, Additional file 2: Fig. S1). To further ensure fasting duration did not affect results, we conducted a sensitivity analysis adjusting for fasting duration and obtained similar results in all models (Additional file 1: Table S9, S10 and S11). Therefore, we are confident that the semi-fasted nature of blood samples did not affect our results. Nonetheless, the self-reported assessment of participants’ fasting duration does represent a limitation, but is unlikely to introduce systematic error. Some variables used for sub-group analysis demonstrated a non-linear relationship with the menstrual cycle phase (fat mass %, fat-free mass %, grip strength, cardiorespiratory fitness). Therefore, results from the sub-group analyses must be interpreted with caution. Data analysed in this study were collected once per participant and are therefore relevant to a single timepoint within their menstrual cycle. Future studies are warranted with serial measurements from the same individual spanning multiple timepoints across the menstrual cycle. Progesterone exerts antagonistic effects to the actions of estradiol on metabolic control [60]. However, in UK Biobank, progesterone was not assessed. Future prospective cohort studies assessing ovarian hormones should endeavour to analyse all ovarian hormones, including progesterone, to facilitate the examination of the metabolic actions of synchronous fluctuations in hormonal profiles.

The large prospective nature of the UK Biobank cohort is a substantial strength of this study. We analysed data from 8694 regularly menstruating women, the largest study to date examining the variation in metabolites across the menstrual cycle. Moreover, detailed information collection on menstrual cycle characteristics allowed the exclusion of women with any symptoms of irregular menstrual cycles and peri-menopausal phase.

## Conclusions

In conclusion, our study confirms previous findings by demonstrating that lipid profiles exhibit non-linear variations by menstrual cycle phase. We did not find evidence that the associations between the menstrual cycle phase and glucose; triglyceride; TyG index; total, HDL and LDL cholesterol; and total to HDL cholesterol were mediated by anthropometric, physical activity, fitness or inflammatory markers. We identified fat mass, physical activity level and cardiorespiratory fitness as factors modifying the association between menstrual cycle and glucose; triglyceride; TyG index; total, HDL and LDL cholesterol; and total to HDL cholesterol. These findings should be considered in therapeutic strategies to mitigate disturbances in metabolic control across the menstrual cycle. Further work is required to examine whether these relationships represent a causal mechanism underpinning variation in metabolic control across the menstrual cycle.

### Supplementary Information

**Additional file 1: Table S1.** Relationship between participant characteristics and menstrual cycle status. **Table S2.** Participant characteristics in the early follicular phase, ovulatory phase and mid-luteal phase. **Table S3.** Effect of anthropometric, lifestyle, fitness and inflammatory markers on the association between metabolite concentration in the early follicular phase, ovulatory phase and mid-luteal phase. **Table S4.** Anthropometric, lifestyle and fitness markers as potential effect modifiers of the association between menstrual cycle phase and metabolites. **Table S5.** Anthropometric, lifestyle and fitness markers as potential effect modifiers of the association between menstrual cycle phase and metabolites in the early follicular phase, ovulatory phase and mid-luteal phase. **Table S6-S8**. Results obtained in the sensitivity analysis for menstrual cycle symptoms. **Table S9-S12**. Results obtained in the sensitivity analysis for fasting duration.

**Additional file 2: Figure S1.** Changes in metabolite concentration across hourly fasting durations.

## Data Availability

UK Biobank data is available to researchers on application (https://www.ukbiobank.ac.uk/enable-your-research).

## Abbreviations

BMI: Body mass index
CRP: C-reactive protein
EDF: Estimated degree of freedom
GAM: Generalised additive model
HBA1c: Glycated haemoglobin
HDL: High-density lipoprotein
IGF-1: Insulin-like growth factor-1
IL-4: Interleukin-4
IPAQ: International Physical Activity Questionnaire
LDL: Low-density lipoprotein
MET: Metabolic energy equivalent
TNF-a: Tumour necrosis-alpha
TyG index: Triglyceride to glucose index
T2D: Type 2 diabetes
