# Supplementary material for: Association between menstrual cycle phase and metabolites in healthy, regularly menstruating women in UK Biobank, and effect modification by inflammatory markers and risk factors for metabolic disease
Source: BMC Med. 2023 Dec 8;21:488. doi: 10.1186/s12916-023-03195-w (PMC10709933; doi:10.1186/s12916-023-03195-w)
Supplement: Supplementary file 2 — Additional file 2: Figure S1. Changes in metabolite concentration across hourly fasting durations. [file 12916_2023_3195_MOESM2_ESM.docx]

**Figure S1:** Changes in metabolite concentration across hourly fasting durations. Data are presented as mean ± 1 SD.
